# Calcitonin gene‐related peptide concentration in cerebrospinal fluid and serum in horses affected by trigeminal‐mediated headshaking

**DOI:** 10.1002/evj.70139

**Published:** 2025-12-19

**Authors:** Lisa Annabel Weber, Hilke Oltmanns, Ludovica Chiavaccini, Kirstie J. Pickles, Veronica Roberts, Tanja Kloock, Tobias Niebuhr, Karsten Feige

**Affiliations:** ^1^ Clinic for Horses, University of Veterinary Medicine Hannover, Foundation Hannover Germany; ^2^ Department of Pharmacology, Toxicology and Pharmacy University of Veterinary Medicine Hannover, Foundation Hannover Germany; ^3^ Department of Comparative Diagnostic and Population Medicine University of Florida Gainesville Florida USA; ^4^ Harper & Keele Veterinary School, Keele Campus Staffordshire UK; ^5^ Bristol Veterinary School, University of Bristol Langford Bristol UK; ^6^ Institute of Functional and Applied Anatomy, Hannover Medical School Hannover Germany; ^7^ Equine Clinic Nindorf Hanstedt Germany

**Keywords:** biomarker, horse, neurology, neuropeptide, peripheral blood, trigeminal neuralgia

## Abstract

**Background:**

Trigeminal‐mediated headshaking (TMHS) in horses shares clinical features with human trigeminal neuralgia (HTN). Increased levels of the neuropeptide calcitonin gene‐related peptide (CGRP) have been found in the blood and cerebrospinal fluid (CSF) of HTN patients. Inhibition of CGRP in humans has shown promise for pain relief. Data on CGRP in horses affected by TMHS are currently lacking but if quantifiable and validated, could assist in developing new diagnostic and more rational therapeutic approaches.

**Objectives:**

This study aimed to quantify and correlate CGRP concentrations in the serum and CSF of horses with TMHS; compare CSF CGRP levels across horses with various neurological, painful, inflammatory and chronic conditions; analyse serum CGRP concentrations before and after exercise‐induced headshaking in affected horses versus healthy controls.

**Study design:**

Case–control study using bio‐banked samples and prospective before‐after study.

**Methods:**

CGRP concentrations were measured in CSF and serum using a commercial ELISA kit across healthy controls (CONTROL, *n* = 5), TMHS‐affected horses (*n* = 30), horses with cervical vertebral stenotic myelopathy (CVSM, *n* = 10) and horses with non‐neurologic painful, inflammatory or chronic conditions (MIXED, *n* = 8).

**Results:**

Median (interquartile range [IQR]) serum CGRP concentration in TMHS horses was 14.9 pg/mL (11.3–19.0 pg/mL), while mean ± SD CGRP CSF concentration was 64.9 ± 6.3 pg/mL. No correlation was found between serum and CSF CGRP (Spearman's rho = −0.04, *p* = 0.88). CSF CGRP levels were significantly higher in CVSM (73.8 pg/mL; *p* = 0.011) and lower in MIXED (52.0 pg/mL; *p* = 0.001) compared to TMHS. Serum CGRP concentrations showed no significant difference between TMHS and CONTROL groups.

**Main Limitations:**

Small sample size, lack of CSF samples from healthy controls.

**Conclusions:**

Higher CSF CGRP levels in neurologic conditions may suggest shared underlying mechanisms such as nerve irritation or neuroinflammation. Further research is needed to elucidate CGRP's role in TMHS pathophysiology.

## 1 INTRODUCTION

Equine trigeminal‐mediated headshaking (TMHS) is a neuropathic facial pain condition clinically characterised by uncontrollable head flicks, muzzle jerking, vertical head‐shaking and signs of nasal irritation, impacting the horses' ability to be ridden or handled.^1^ Despite its impact on equine welfare, the exact aetiopathogenesis of TMHS has not been elucidated.^1^, ^2^ Due to yet unknown causes, a decreased activation threshold of the trigeminal nerve occurs, causing it to be sensitised and fire spontaneously or with minimal stimulus (e.g., wind, sun light, exercise, touching the horses' nose).^3^, ^4^, ^5^ The condition appears to resemble human trigeminal neuralgia in certain clinical aspects.^2^

Human trigeminal neuralgia (HTN) is characterised by sudden, brief or sustained, excruciating facial pain in the trigeminal nerve area, which patients describe as itching, tingling, burning and electric shock‐like sensations.^6^, ^7^ The disease is extremely debilitating in people, leading to a severe reduction in the quality of life of affected patients.^6^ Due to the lowered threshold for nerve firing and the clinical signs shown in horses affected by TMHS, it is rational to assume they also experience significant neuropathic pain and distress.^2^, ^8^ There is no single, universally safe and curative treatment for TMHS. Current interventions primarily consist of medical therapies or the use of neuromodulatory devices.^1^, ^9^, ^10^, ^11^ The former may involve side effects and doping restrictions, while the latter often provides only temporary relief, with reported efficacy and recurrence rates varying in the literature and not always being directly comparable.^1^, ^2^, ^9^, ^10^ Some horses have to be retired early or euthanased due to the disease.^1^, ^11^, ^12^, ^13^ The clinical diagnosis of TMHS is based on a relatively complex and expensive exclusion of differential diagnosis.^8^, ^13^, ^14^, ^15^ Biomarkers of neuropathic pain are yet to be identified in such affected horses.^1^, ^12^, ^14^, ^16^

Calcitonin gene‐related peptide (CGRP) is a potent neuropeptide widely distributed in nociceptive pathways in the human peripheral and central nervous system (CNS).^15^, ^17^ Trigeminal nerve activation releases CGRP, which acts as a facilitator in pain transmission and contributes to generating a hyper‐responsive state.^6^, ^7^ As patients with HTN show increased CGRP concentrations in both blood and cerebrospinal fluid (CSF), the peptide has gained interest as a potential peripheral biomarker^18^, ^19^, ^20^ and therapeutic target in patients affected by this condition^6^, ^7^, ^21^, ^22^ and migraine.^23^, ^24^, ^25^ Data on CGRP in horses affected by TMHS are currently lacking but, if quantifiable and validated, could assist in developing new diagnostic and more rational therapeutic approaches.

To investigate whether the upregulation of the excitatory neuropeptide CGRP is a pathogenic component of TMHS and to elucidate a potential biomarker for the condition, the first aim of this study was to quantify and correlate the expression of CGRP in the CSF and peripheral blood of horses affected by the disease. The second aim was to compare CSF CGRP concentrations in TMHS and horses affected by different neurologic, painful, inflammatory or chronic conditions. As CGRP concentrations may increase only transiently during an acute episode of exercise‐induced headshaking,^20^, ^26^, ^27^ the third aim was to analyse the serum concentrations of CGRP in healthy horses and TMHS‐affected horses at rest and immediately after exercise.

## 2 MATERIALS AND METHODS

### 2.1 Animals

Prospectively collected and bio‐banked serum and CSF samples of 53 horses presented to the Clinic for Horses of the University of Veterinary Medicine Hannover, Foundation, Hannover, Germany, were used. Samples were obtained between 2018 and 2024. The number of animals included in this study was determined by convenience sampling based on the availability of relevant biobanked samples and the presentation of suitable cases in the study period. Horses were either diagnosed with trigeminal‐mediated headshaking (TMHS, *n* = 30; this population is divided into TMHS‐1&2 used in Aims 1 and 2, *n* = 16, and TMHS‐3 used in Aim 3, *n* = 14), cervical vertebral stenotic myelopathy (CVSM, *n* = 10) or other diseases (MIXED, *n* = 8) including small intestine strangulation due to strangulating lipoma (*n* = 2), severe cystitis (*n* =1), metastatic adenocarcinoma of the urinary bladder with severe cystitis (*n* = 1), laminitis (*n* =1), atrial fibrillation with syncope (*n* = 1), sepsis (*n* = 1), persistent postoperative ileus after colon torsion (*n* = 1) or healthy controls (CONTROL, *n* = 5).

### 2.2 Diagnosis of TMHS and scoring of headshaking

The diagnosis of TMHS was established by exclusion of other causes for (secondary) headshaking. A standardised, modified protocol was applied^12^ which included history, clinical, neurologic, orthopaedic and ophthalmologic examinations, complete blood work, CSF analysis, computer tomography (CT) of the head and, when indicated, CT imaging of the neck. Additional diagnostics, such as case‐dependent local anaesthesia of suspected head regions, were performed as needed. Since a standing CT scanner was unavailable at the study facility during the study period, the CT examinations were conducted under general anaesthesia. Disease severity was assessed using the History, Rest and Exercise Score.^28^ For horses in the TMHS‐1&2 group, if multiple headshaking scores were recorded in the clinical records for either rest or exercise, the average score was calculated for each sub‐score. Horses in the groups TMHS‐3 and CONTROL were scored during a standardised exercise test (see below), with rest scores obtained earlier on the same day. The total headshaking score for each horse was determined as the average of the history, rest and exercise scores.^28^

### 2.3 Sample collection

CSF and serum samples of TMHS‐1&2 and CSF from CVSM were collected as part of the routine diagnostic workup of each horse. The analysis of these samples did not require ethical approval. CSF samples from the MIXED group, used for Aim 2, were obtained immediately after euthanasia. Euthanasia was performed for medical reasons unrelated to this study. Serum samples from TMHS‐3 and CONTROL groups, used for Aim 3, were collected as part of a separate, unpublished study.

Blood samples were withdrawn by jugular vein venepuncture. Blood was allowed to clot at room temperature and centrifuged at 3000 rounds per minute for 6 min. Serum was collected and stored at −70°C or −80°C until further analysis.

CSF was collected via atlanto‐occipital puncture of the sub‐arachnoid space, either during the same general anaesthesia under which the CT scan was performed, with the horse positioned in lateral recumbency (TMHS‐1&2, CVSM), or following euthanasia with the horse placed in lateral or dorsal recumbency (MIXED). The samples were taken aseptically from TMHS‐1&2 and CVSM. The collected CSF samples of TMHS‐1&2 and MIXED were centrifuged for 15 min at 10,000*g*. No information about centrifugation of CVSM CSF samples was available. CSF samples were stored at −70°C or −80°C until further analysis.

The time of sample collection was recorded for the prospectively collected samples (CSF of MIXED, serum of TMHS‐3 and CONTROL) or estimated for the retrospective samples (serum and CSF of TMHS‐1&2, CSF of CVSM) from documentation and on the basis of standardised procedures in the clinic. The duration for which the samples were stored was also recorded.

### 2.4 Exercise test

To compare serum CGRP concentrations in TMHS‐affected horses and healthy control horses after a standardised exercise test (Aim 3), a basal serum sample was withdrawn in the morning at rest. Horses were then lunged for 10 min at trot and 5 min at canter. Immediately after the exercise test, a second serum sample was obtained.

### 2.5 Enzyme‐linked immunosorbent assay

To determine the concentrations of CGRP in serum and CSF, a species‐validated, commercial two‐site sandwich ELISA kit (MyBioSource) was utilised according to manufacturer's instructions. In brief, an antibody specific to CGRP was pre‐coated onto the microplate. Hundred microliters of standard or sample was pipetted into the wells and incubated for 2 h at 37°C. After removing any unbound substances, 100 μL of a biotin‐conjugated antibody specific for CGRP was added to each well and incubated for 1 h at 37°C. After washing, 100 μL streptavidin‐conjugated horseradish peroxidase was added to each well and incubated for 1 h at 37°C. Following washing to remove any unbound avidin‐enzyme reagent, 100 μL of substrate solution was added to the wells and incubated for 15–20 min at 37°C. Colour developed proportionately to the amount of CGRP bound in the initial step. The optical density of each well was measured within 5 min by a microplate reader capable of measuring absorbance at 450 nm, with the correction wavelength set at 540 nm.

### 2.6 Data analysis

All statistical analyses were performed with Stata/BE 17.0 for Mac (StataCorp LLC). Continuous data were checked for normality distribution using the Shapiro–Wilk test and graphically using the histogram and the normal quantile distribution in Stata. Normally distributed data were presented as mean ± SD and non‐normally distributed data were reported as median (interquartile range [IQR]). Spearman's rank correlation coefficient was used to evaluate associations between CSF and serum CGRP concentrations, headshaking scores in TMHS‐1 horses and age in TMHS‐2 horses. To compare CSF CGRP concentrations among horses with TMHS, CVSM and other conditions, two separate analyses were performed: a one‐way ANOVA with Bonferroni correction for multiple comparisons and a linear regression model, including age as a covariate to account for its potential confounding effect. The CSF CGRP concentrations were also compared among sexes using a one‐way ANOVA. Serum CGRP concentrations between TMHS‐affected horses (TMHS‐3) and control horses were compared at rest using the Mann–Whitney *U*‐test. Then data were rank transformed and a mixed effect linear regression model on ranks was used to evaluate the effects of condition (TMHS vs. control), time (pre‐ vs. post‐exercise) and their interaction (fixed effects) on serum CGRP, with horses modelled as random intercepts. All analyses were two‐tailed, and statistical significance was defined as *p* ≤ 0.05 unless otherwise specified.

## 3 RESULTS

### 3.1 Animals

The signalment of the horses and details of the samples analysed for CGRP concentration are summarised in Table 1.

**TABLE 1:** Signalment and analysed samples of the study population.

	CONTROL (*n* = 5)	TMHS‐1&2 (*n* = 16)	TMHS‐3 (*n* = 14)	CVSM (*n* = 10)	MIXED (*n* = 8)
Age	5.0 (5.0–9.0)	9.0 (7.0–10.5)	8.0 (7.0–13.0)	5.5 (3.0–8.0)	18.5 (13.0–23.0)
Sex	Mares (*n* = 1)	Mares (*n* = 6)	Mares (*n* = 5)	Mares (*n* = 3)	Mares (4)
	Geldings (*n* = 4)	Geldings (*n* = 9)	Geldings (*n* = 9)	Geldings (*n* = 2)	Geldings (4)
		Stallions (*n* = 1)		Stallions (*n* = 5)	
Breeds	Hanoverian WB (*n* = 2)	Hanoverian WB (*n* = 4)	German Sport Horse (*n* = 3)	Hanoverian WB (*n* = 3)	Hanoverian WB (*n* = 2)
	Oldenburger WB (*n* = 2)	Westphalian WB (*n* = 3)	Hanoverian WB (*n* = 2)	Oldenburger WB (*n* = 2)	Oldenburger WB (*n* = 1)
	Irish Sport Horse (*n* = 1)	Oldenburger WB (*n* = 2)	Westphalian WB (*n* = 2)	English Thoroughbred (*n* = 2)	Mecklenburger WB (*n* = 1)
		Mecklenburger WB (*n* = 1)	Rhenish WB (*n* = 1)		
					Westphalian WB (*n* = 1)
		Rhenish WB (*n* = 1)	Dutch WB (*n* = 1)	Danish WB (*n* = 1) Westphalian WB (*n* = 1)	Polish WB (*n* = 1)
		Baden‐Wuerttemberger WB (*n* = 1)	Oldenburg WB (*n* = 1)		Welsh A Pony (*n* = 1)
		Quarter Horse (*n* = 1)	Polish WB (*n* = 1)	Selle Français (*n* = 1)	Anglo‐Arabian (*n* = 1)
			Appaloosa (*n* = 1)		
		German Riding Pony (*n* = 1)	Zangersheide (*n* = 1)		
		German Sport Horse (*n* = 1)	American Quarter Horse (*n* = 1)		
		Unknown (*n* = 1)			
Samples analysed	Serum collected at rest and after exercise	Serum collected at rest and CSF withdrawn under general anaesthesia	Serum collected at rest and after exercise	CSF withdrawn under general anaesthesia	CSF withdrawn after euthanasia

*Note:* Data are presented as numbers or median (interquartile range [IQR]).

Abbreviations: CONTROL, healthy control horses; CSF, cerebrospinal fluid; CVSM, cervical vertebral stenotic myelopathy; MIXED, horses with different diseases; *n*, number; TMHS, trigeminal‐mediated headshaking; WB, warmblood.

### 3.2 Aim 1: Quantification and correlation of CGRP in peripheral blood (serum) and CSF in TMHS‐affected horses

Serum samples were collected between 9:00 AM and 10:00 AM, while CSF samples were collected between 8:30 AM and 2:00 PM, with the majority (13/16) collected between 8:30 and 9:30 a.m. Serum and CSF samples were stored for a median (IQR) of 8.5 months (2.8–14). The median (IQR) scores for history, rest and exercise were 100% (83–100%), 12% (6.8–24.6%) and 25.9% (20–37%), respectively, with a total score of 46.8% (41–53.8%). The median (IQR) serum CGRP concentration was 14.9 pg/mL (11.3–19.0 pg/mL), while the mean ± SD CGRP concentration in the CSF was 64.9 ± 6.3 pg/mL. Serum and CSF CGRP concentrations exhibited a negligible negative correlation (Spearman's rho = −0.04, *p* = 0.88). A significant moderate correlation was observed between CSF CGRP concentrations and both the TMHS history score (Spearman's rho = −0.54, *p* = 0.036) and the rest score (Spearman's rho = 0.62, *p* = 0.030). However, there was no correlation between CSF concentrations of CGRP and severity of TMHS as defined by exercise (Spearman's rho = −0.14, *p* = 0.64) and total scores (Spearman's rho = 0.04, *p* = 0.89).

### 3.3 Aim 2: Comparison of CSF CGRP concentrations in horses affected by TMHS with horses affected by different neurologic, painful or inflammatory conditions

CSF samples in the CVSM group were withdrawn between 9:00 AM and 2:00 PM, with the majority (8/10) collected before 11 AM. Samples of MIXED were obtained between 11:45 AM and 6:30 PM. The samples of the CVSM group were stored for a median (IQR) of 51.5 (22.3–58.5) months, while the samples of the MIXED group were stored for 2 (1–11.8) months.

**FIGURE 1:**
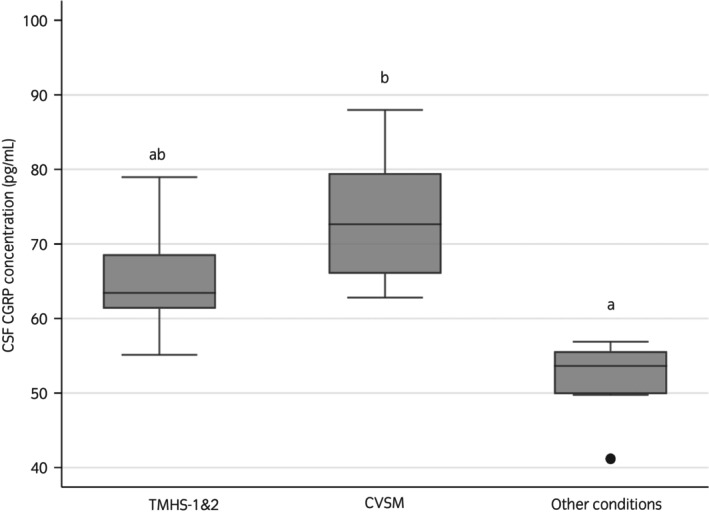
Box‐plot representing the cerebrospinal fluid calcitonin‐gene related peptide (CGRP) concentrations (in pg/mL) in 16 horses affected by trigeminal‐mediated headshaking (TMHS‐1&2), 10 horses affected by cervical vertebral stenotic myelopathy (CVSM) and 8 horses euthanised for other painful, inflammatory or chronic conditions. Group comparisons were performed using a one‐way ANOVA followed by Bonferroni correction for multiple comparisons. Superscript letters indicate statistically significant differences: (a) TMHS versus other conditions, *p* = 0.011. (b) TMHS versus CVSM, *p* = 0.001.

The mean ± SD concentration of CGRP in the CSF of horses affected by CVSM was 73.8 ± 9.1 pg/mL, which was significantly higher than in the TMHS group (*p* = 0.011) (Figure 1). In contrast, the mean ± SD concentration of CGRP in the CSF of horses euthanised for other conditions was 52.0 ± 5.1 pg/mL, significantly lower than that in the TMHS group (*p* = 0.001) (Figure 1). A moderate negative association was observed between age and CSF CGRP concentrations (Spearman's rho = −0.54, *p* = 0.001); however, age lost statistical significance in a multivariable linear regression model, suggesting some collinearity with the condition. No association was found between sex and CSF CGRP concentrations (*p* = 0.59). Macroscopic (all groups) and laboratory analysis (TMHS and CVSM) of CSF samples excluded blood contamination's impact on CGRP results.

### 3.4 Aim 3: Serum concentrations of CGRP in control horses and TMHS‐affected horses before and after a standardised exercise test

All serum samples before and after exercise were collected between 9:00 and 9:30 AM. Affected horses had a median (IQR) age of 8 years (7–13 years), while control horses had a median (IQR) age of 5 years (5–9 years) with no statistically significant evidence of an age difference between groups (*p* = 0.18). For control horses, the median (IQR) scores for history, rest and exercise were 0% (0–0%), 4.2% (0–4.2%) and 6.7% (6.7–10.8), respectively, with a total score of 3.6% (2.2–4.7%). In contrast, TMHS‐affected horses had a median (IQR) history score of 87.5% (80–90%) and rest score of 10.4% (5.5–12.5%). All TMHS‐affected horses displayed headshaking signs during the exercise test with a median (IQR) exercise score of 33% (28.3–36.7%). The total score in TMHS‐3 was 43.5% (39.5–48.2%).

A 9‐year‐old Oldenburg Warmblood gelding in the control group recorded a CGRP serum concentration at rest of 1199.9 pg/mL that decreased after exercise to 1140.5 pg/mL. This was identified as an outlier and excluded from further analysis.

**FIGURE 2:**
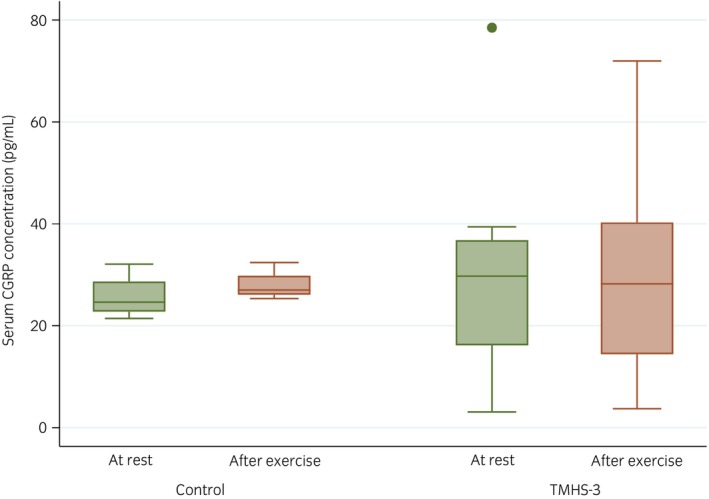
Box‐plot representing the serum calcitonin‐gene related peptide (CGRP) concentration (expressed in pg/mL) in four healthy control horses and 14 horses affected by trigeminal‐mediated headshaking (TMHS‐3), before and after light exercise.

**FIGURE 3:**
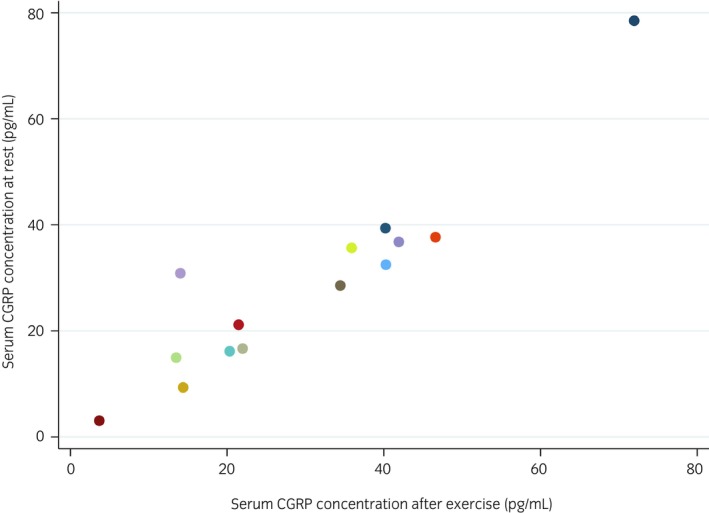
A scatter plot illustrating the correlation between serum CGRP concentrations measured before and after exercise in 14 horses affected by trigeminal‐mediated headshaking (TMHS‐3). Each data point represents an individual horse. Despite a broad range of CGRP values across the group, most points are located near the line of identity (the diagonal), indicating that the levels for individual horses changed very little with exercise.

With the outlier excluded, the final median (IQR) CGRP serum concentrations at rest were 24.6 pg/mL (22.7–28.7 pg/mL) in the control group and 29.7 pg/mL (16.2–36.8 pg/mL) in the TMHS‐3 group, with no statistically significant difference between the two groups (*z* = −0.21, *p* = 0.88). After exercise, the median (IQR) CGRP serum concentrations were 27.2 pg/mL (26.9–32.4 pg/mL) in the control group and 28.2 pg/mL (14.39–40.27 pg/mL) in the TMHS‐3 group. No significant difference in CGRP concentrations was found between TMHS‐affected horses and controls before or after exercise (Wald *χ* ^2^ = 2.47, *p* = 0.48) (Figure 2). Although CGRP values showed notable variability among horses with headshaking, individual animals exhibited minimal changes in CGRP concentrations before and after exercise (Figures 2 and 3).

## 4 DISCUSSION

Progress in diagnosing and treating TMHS in horses will remain restricted until the condition's aetiology and pathogenesis are better understood. This study therefore investigated CGRP in such affected horses, a neuropeptide that plays a major role in the pathophysiology of orofacial pain and headache in humans and other species.^7^, ^29^, ^30^ CSF CGRP levels were significantly increased in horses with TMHS and CVSM compared to those euthanised for other non‐neurologic painful, inflammatory or chronic conditions. These findings suggest that increased CSF CGRP concentrations do not serve as a specific biomarker for TMHS but could be associated with neuropathic pain and/or neuropathic inflammation in horses. No correlation was identified between CGRP serum and CSF concentrations in horses with TMHS. Furthermore, no link was found between acute exercise‐related headshaking and increased serum CGRP levels.

Calcitonin gene‐related peptide is involved in the transmission of both central and peripheral pain as well as the inflammatory response, particularly, but not exclusively in conditions affecting the nervous system.^7^ Activation of the trigeminal nerve system induces the release of CGRP in mice, rats, cats and humans.^7^, ^31^ The neuropeptide enhances pain signalling, contributes to the development of a hyperresponsive state and causes cerebral vascular vasodilation.^7^, ^31^ Increased CSF CGRP levels in horses with TMHS likely indicate and support previous assumptions of an underlying neuropathic pain mechanism,^1^, ^2^ with potential involvement of trigeminal nerve dysfunction and central sensitisation. These findings suggest that TMHS may be associated with neuropathic pain in horses and open the door to the development of new therapeutic approaches for this condition, such as CGRP antagonists or monoclonal antibodies.

The concentrations of CGRP in serum and CSF of horses with TMHS exhibited a negligible negative correlation, suggesting that CGRP concentrations in serum do not reliably reflect CGRP levels in the CNS in these horses. This is in contrast to patients with HTN and migraine, in which CGRP was found to correlate in peripheral blood and CSF.^19^, ^32^ Calcitonin gene‐related peptide levels may be connected between CSF and serum in humans but not in horses due to species‐specific differences in blood–brain barrier permeability, CGRP metabolism or neurovascular anatomy. Specific data regarding this is not available for horses. Serum CGRP levels of healthy horses were studied within a master thesis.^33^ The reported mean serum CGRP concentrations of 34.5 pg/mL in healthy geldings and 25.8 pg/mL in healthy foals are comparable to the blood values of the present control and headshaking horse population. However, due to the less than 100% recovery rate of the rat CGRP‐ELISA used, the author of the aforementioned study speculated that the actual concentration of CGRP in equine plasma is likely higher.^33^ In healthy humans, serum CGRP values were 2 pg/mL,^34^ 11.4 pg/mL^35^ and >500 pg/mL.^36^ Inconsistent data about CGRP concentrations in human blood of healthy individuals have been attributed to different analysis methods.^36^ For the purposes of future equine studies, it seems reasonable to utilise the same analysis method for comparison. While a circadian profile of CGRP in the blood has been described in healthy and diseased humans,^37^, ^38^ this has not been demonstrated in healthy mares and foals with signs of systemic inflammatory response syndrome.^33^ Even though the analysis of a circadian rhythm of the neuropeptide was not an aim of this study, details on sampling time points in the materials and methods section were provided to ensure reproducibility, transparency and to enable future studies to build upon this work.

The study demonstrated a correlation between history and rest scores and CSF CGRP levels. In contrast, no association was observed between CSF CGRP concentrations and signs of headshaking during exercise. Horses exhibiting headshaking at rest are assumed to demonstrate significant compromised welfare, as they appear to experience pain and discomfort even during periods when they should recover.^1^ A recent study showed that horses severely affected by TMHS experience pain even at rest between headshaking episodes.^8^ The severity of clinical signs during rest and the duration of the horse's disease are not correlated with the severity of clinical signs during exercise.^39^ One could speculate that a longer disease duration may lead to greater sensitisation within the central nervous system, which is either caused by or results in higher CGRP values. The findings could further indicate the importance of assessing the signs of affected horses using a dual approach, both during exercise and rest. While the severity of pain symptoms in patients with HTN was correlated with CGRP levels,^19^ the actual relationship between neuropeptide value and pain intensity associated with TMHS must be elucidated. Conversely to our findings, CGRP levels in HTN were unrelated to the duration of the disease.^19^

Calcitonin‐gene related peptide is present in the dorsal and ventral horns of the spinal cord, as well as in the dorsal root ganglia of horses.^40^, ^41^ Therefore, the observed increased CGRP concentrations in the CSF of horses with CVSM may indicate ongoing nerve irritation/damage or neuroinflammation within the CNS. In these cases, inflammation of the nervous tissue is thought to result from chronic or intermittent compression and/or stenosis of the vertebral canal, causing repeated trauma.^42^, ^43^ Studies in rats and humans have shown that CGRP immuno‐positive fibres show sprouting within the dorsal horn following spinal cord injury.^44^, ^45^ No reliable statement can be made about the pain level of the CVSM horses examined, as these CSF samples were analysed retrospectively. In contrast to older horses, clinical signs of cervical pain rarely appear in young‐aged CVSM‐affected horses.^42^, ^43^ The CVSM population in this study was rather young and, therefore, an association between pain and increased CGRP levels is questionable. The elevation of CGRP in both neurologic groups (TMHS and CVSM) has not been described in horses yet. It could be a suggestion that there are similar underlying processes like nerve irritation, enhanced neuropathic pain or neuroinflammation, rather than a disease‐specific biomarker. This study can serve as a basis for future studies related to these and other neurologic conditions.

The horses within the MIXED group displayed a wide array of acute and chronic visceral and somatic painful or inflammatory conditions. Remarkably, despite this diversity of diseases, no elevation in CSF CGRP levels was observed in this group compared to horses with TMHS or CVSM. Currently, there is no data available regarding the stability of equine CGRP in serum or CSF samples. Long‐term storage of human plasma and serum samples decreases the concentration of CGRP.^36^, ^46^ Since the CSF samples from the MIXED group were stored for a shorter duration compared to the others, the lower levels of CGRP observed in this group compared to the higher levels in the TMHS or CVSM groups cannot be explained by reduced stability resulting from prolonged freezing. The CSF samples from the CVSM horses, which had the highest CGRP concentration, were stored for the longest time. Still, future research on CGRP in horses should investigate the long‐term storage stability of the peptide to adjust the design of subsequent studies if necessary and make data interpretation easier. Unfortunately, CSF from healthy horses was not available for CGRP quantification, which is a main limitation of this study. In human visceral pain conditions, only gynaecological pain correlated with high CGRP levels in tissue biopsies and peritoneal fluid, while no correlation was found in painful cardiovascular or gastrointestinal diseases.^15^ Apart from a headache, an intravenous CGRP infusion did not cause any other pain in migraine sufferers.^24^ No differences in CGRP concentrations were found in the CSF between human cancer patients and controls.^15^ Additionally, patients with chronic orthopaedic pain exhibited decreased CSF CGRP levels compared to healthy controls.^47^ As in humans, the low CSF CGRP levels in the MIXED group might imply that CGRP is not universally increased in all forms of pain or inflammation in horses. Visceral and somatic pain pathways in horses seem not to be closely linked to CGRP in the CSF.

Horses with TMHS often exhibit pronounced headshaking signs during exercise.^1^, ^48^ The mechanism by which movement triggers headshaking in these horses is unknown. Despite all TMHS‐affected horses in this study demonstrating headshaking during the exercise test, the serum CGRP concentrations remained consistent and comparable to those of healthy controls, both before and after exercise. Acute migraine and cluster headache attacks in people have been linked in multiple studies to increased CGRP levels in jugular blood,^26^, ^27^ and chronic migraineurs show raised concentrations of the neuropeptide even in interictal phases.^32^, ^49^ In people, the concentration of CGRP in serum significantly increases with medium‐distance endurance exercise, and the CGRP post‐exercise rise depends on the intensity of running, suggesting a role in exertional headache and migraine.^20^ It remains therefore speculative why this study did not find a correlation between acute exercise‐associated headshaking and increased levels of CGRP. First, the analysis of serum and CSF values in the TMHS population investigated in Aim 1 showed no correlation. Second, it is possible that the chosen exercise intensity was not sufficient to induce a noticeable increase in the neuropeptide. Finally, acute, exercise‐induced headshaking may not be linked to serum CGRP levels at all. Understanding these dynamics could lead to deeper insights into the pathophysiological responses to exercise and the mechanisms that underlie headshaking.

The study has several limitations that may influence the interpretation and the general use of the findings. First, the small sample size in each group may not represent the broader population. Additionally, the correlation of CGRP in serum and CSF of TMHS‐1 must be evaluated in light of the fact that the samples were taken at approximately the same time in the morning but not on the same day. Blood samples obtained from untreated HTN patients 1 day apart showed no difference in CGRP levels.^19^ A correlation of the peptide in CSF and serum was not possible in the CVSM and MIXED group, as no serum samples were available from these horses. CSF samples were collected either under general anaesthesia (TMHS‐1 and CVSM) or after euthanasia (MIXED). No data are available on the extent to which CGRP levels change in horses under these conditions. As key effects of anaesthetics are the suppression of pain signalling in the central nervous system, an influence on CGRP levels is possible. Due to ethical reasons, general anaesthesia only for the purpose of CSF sampling was not conducted in healthy horses, resulting in the absence of corresponding values. Future study designs could include CSF collection from the C1 to C2 or lumbosacral space in standing, sedated horses if ethically suitable. This technique would also exclude a potential effect of general anaesthesia on the protein. Finally, due to the in‐part retrospective nature of the study, detailed information on headshaking behaviour in horses from the MIXED and CVSM groups was unavailable, and few headshaking scoring data were missing in the TMHS‐1 group. However, no headshaking behaviour was recorded in the anamnesis records of MIXED and CVSM or neurologic examination protocols of CVSM.

In summary, this study suggested that CGRP may be involved in the pathophysiology of TMHS. Increases in the neuropeptide in CSF were observed in both horses with TMHS and CVSM, but not in those with other painful, inflammatory or chronic diseases. As such, CSF CGRP elevations may be associated with processes such as nerve irritation, neuropathic pain or neuroinflammation, but do not yet differentiate aetiology or serve as a TMHS‐specific biomarker. No correlation was found between serum and CSF CGRP levels in horses with TMHS, reducing the clinical value of measuring CGRP in the peripheral blood. Additionally, no link was identified between acute exercise‐related headshaking and increased serum CGRP levels. Further research is needed to clarify the neuropeptides' exact pathophysiological role in this complex disease.

## FUNDING INFORMATION

Not applicable.

## CONFLICT OF INTEREST STATEMENT

The authors declare no conflict of interests.

## AUTHOR CONTRIBUTIONS

**Lisa Annabel Weber:** Investigation; writing – original draft; formal analysis; project administration; conceptualization; writing – review and editing. **Hilke Oltmanns:** Investigation; formal analysis; methodology. **Ludovica Chiavaccini:** Writing – review and editing; visualization; formal analysis. **Kirstie J. Pickles:** Conceptualization; writing – review and editing. **Veronica Roberts:** Conceptualization; writing – review and editing. **Tanja Kloock:** Investigation; writing – review and editing. **Tobias Niebuhr:** Conceptualization; supervision. **Karsten Feige:** Conceptualization; supervision.

## DATA INTEGRITY STATEMENT

Lisa Annabel Weber and Ludovica Chiavaccini had full access to all the data in the study and take responsibility for the integrity of the data and the accuracy of the data analysis.

## ETHICAL ANIMAL RESEARCH

This study was approved by the animal welfare officer of the University of Veterinary Medicine Hannover, Foundation and the State Office for Consumer Protection and Food Safety (LAVES Reference number: 20/3491).

## INFORMED CONSENT

Consent for sampling and sample banking was obtained from the owners.

## Data Availability

The data that support the findings of this study are openly available in Zenodo at https://doi.org/10.5281/zenodo.17872101.

## References

[1] Roberts V . Trigeminal‐mediated headshaking in horses: prevalence, impact, and management strategies. Vet Med. 2019;10:1–8. 10.2147/vmrr.s163805 PMC6330979 30666296

[2] Pickles K , Madigan J , Aleman M . Idiopathic headshaking: Is it still idiopathic? Vet J. 2014;201:21–30. 10.1016/j.tvjl.2014.03.031 24821361

[3] Aleman M , Rhodes D , Williams DC , Guedes A , Madigan JE . Sensory evoked potentials of the trigeminal nerve for the diagnosis of idiopathic headshaking in a horse. J Vet Intern Med. 2014;28:250–253. 10.1111/jvim.12237 24428325 PMC4895556

[4] Aleman M , Williams DC , Brosnan RJ , Nieto JE , Pickles KJ , Berger J , et al. Sensory nerve conduction and somatosensory evoked potentials of the trigeminal nerve in horses with idiopathic headshaking. J Vet Intern Med. 2013;27:1571–1580. 10.1111/jvim.12191 24107198

[5] Nessler JN , Delarocque J , Kloock T , Twele L , Neudeck S , Meyerhoff N , et al. Sensory nerve conduction stimulus threshold measurements of the infraorbital nerve and its applicability as a diagnostic tool in horses with trigeminal‐mediated headshaking. BMC Vet Res. 2024;20: 1–11. 10.1186/s12917-024-04068-x 38750534 PMC11097574

[6] Gambeta E , Chichorro JG , W. Zamponi G . Trigeminal neuralgia: an overview from pathophysiology to pharmacological treatments. Mol Pain. 2020;16: 1–18. 10.1177/1744806920901890 PMC6985973 31908187

[7] Retsky R , Ashina S , Oved D , Sharon R . Calcitonin gene – related peptide and trigeminal neuralgia. SN Compr Clin Med. 2023;5:75. 10.1007/s42399-023-01407-1

[8] Franzen V , Reisbeck D , Leibl Y , Schoster A , May A . Pain assessment of horses with trigeminal‐mediated headshaking (TMHS) at rest between episodes. J Vet Intern Med. 2025;39: 1–9. 10.1111/jvim.70064 PMC11960477 40168040

[9] Dunkel B , Hildon GL , Coumbe KM , Busuttil E , Von Schweinitz D . Electroacupuncture as a treatment for suspected trigeminal nerve‐mediated head‐shaking in 42 horses. Equine Vet Educ. 2025;37:647–653. 10.1111/eve.14135

[10] Roberts VLH , Bailey M , Patel NK . The safety and efficacy of neuromodulation using percutaneous electrical nerve stimulation for the management of trigeminal‐mediated headshaking in 168 horses. Equine Vet J. 2020;52:238–243. 10.1111/evj.13174 31461784 PMC7317358

[11] Newton SA , Knottenbelt DC , Eldridge PR . Headshaking in horses: possible aetiopathogenesis suggested by the results of diagnostic tests and several treatment regimes used in 20 cases. Equine Vet J. 2000;32:208–216. 10.2746/042516400776563617 10836475

[12] Kloock T , Hellige M , Kloock A , Feige K , Niebuhr T . Impact of different diagnostic procedures on diagnosis, therapy, and outcome in horses with headshaking: recommendations for fast‐track advanced diagnostic and therapeutic protocols. Animals. 2022;12:1–14. 10.3390/ani12223125 PMC9686903 36428354

[13] Ross SE , Murray JK , Roberts VLH . Prevalence of headshaking within the equine population in the UK. Equine Vet J. 2018;50:73–78. 10.1111/evj.12708 28608565

[14] Pickles K . Trigeminal‐mediated headshaking: a diagnostic challenge. Equine Vet Educ. 2022;35(4):195–196. 10.1111/eve.13723

[15] Schou WS , Ashina S , Amin FM , Goadsby PJ , Ashina M . Calcitonin gene‐related peptide and pain: a systematic review. J Headache Pain. 2017;18(1):34. 10.1186/s10194-017-0741-2 28303458 PMC5355411

[16] Fairburn AJ , Meehan LJ , Roberts VLH . Computed tomographic findings in 101 horses presented for the investigation of headshaking. Equine Vet Educ. 2022;35(4):e339–e345. 10.1111/eve.13718

[17] Messlinger K . The big CGRP flood – sources, sinks and signalling sites in the trigeminovascular system. J Headache Pain. 2018;19(1):22. 10.1186/s10194-018-0848-0 29532195 PMC5847494

[18] Zhang Y , Lian Y , Zhang H , Xie N , Chen Y . CGRP plasma levels decrease in classical trigeminal neuralgia patients treated with botulinum toxin type A: a pilot study. Pain Med. 2020;21:1611–1615. 10.1093/PM/PNAA028 32167549

[19] Qin Z , Yang L , Li N , Yue J , Wu B , Tang Y , et al. Clinical study of cerebrospinal fluid neuropeptides in patients with primary trigeminal neuralgia. Clin Neurol Neurosurg. 2016;143:111–115. 10.1016/j.clineuro.2016.02.012 26918582

[20] Tarperi C , Sanchis‐Gomar F , Montagnana M , Danese E , Salvagno GL , Gelati M , et al. Effects of endurance exercise on serum concentration of calcitonin gene‐related peptide (CGRP): a potential link between exercise intensity and headache. Clin Chem Lab Med. 2020;58:1707–1712. 10.1515/cclm-2019-1337 32286239

[21] Schott Andersen AS , Maarbjerg S , Noory N , Heinskou TB , Forman JL , Cruccu G , et al. Safety and efficacy of erenumab in patients with trigeminal neuralgia in Denmark: a double‐blind, randomised, placebo‐controlled, proof‐of‐concept study. Lancet Neurol. 2022;21:994–1003. 10.1016/S1474-4422(22)00294-0 36113495

[22] Parascandolo E , Levinson K , Rizzoli P , Sharon R . Efficacy of erenumab in the treatment of trigeminal neuralgia: a retrospective case series. Neurol Clin Pract. 2021;11:227–231. 10.1212/CPJ.0000000000001075 34484889 PMC8382339

[23] Russo AF . CGRP: a new target for migraine. Annu Rev Pharmacol Toxicol. 2015;60:307–322. 10.1146/annurev-pharmtox-010814-124701.C PMC4392770 25340934

[24] Villalón CM , Olesen J . The role of CGRP in the pathophysiology of migraine and efficacy of CGRP receptor antagonists as acute antimigraine drugs. Pharmacol Ther. 2009;124:309–323. 10.1016/j.pharmthera.2009.09.003 19796656

[25] Oliveira R , Gil‐Gouveia R , Puledda F . CGRP‐targeted medication in chronic migraine – systematic review. J Headache Pain. 2024;25:51. 10.1186/s10194-024-01753-y 38575868 PMC10996229

[26] Goadsby PJ , Edvinsson L . Human in vivo evidence for trigeminovascular activation in cluster headache neuropeptide changes and effects of acute attacks therapies. Brain. 1994;117:427–434. 10.1093/brain/117.3.427 7518321

[27] Iyengar S , Johnson KW , Ossipov MH , Aurora SK . Review articles CGRP and the trigeminal system in migraine. Headache. 2019;59(5):659–681. 10.1111/head.13529 30982963 PMC6593989

[28] Kloock T , Pickles KJ , Roberts V , Uhlendorf F , Twele L , Wilkens HL , et al. History, Rest and Exercise Score (HRE‐S) for assessment of disease severity in horses with trigeminal‐mediated headshaking. Equine Vet J. 2024;56:464–474. 10.1111/evj.13986 37608443

[29] Afroz S , Arakaki R , Iwasa T , Oshima M , Hosoki M , Inoue M , et al. CGRP induces differential regulation of cytokines from satellite glial cells in trigeminal ganglia and orofacial nociception. Int J Mol Sci. 2019;20(3):711. 10.3390/ijms20030711 30736422 PMC6386987

[30] Romero‐Reyes M , Pardi V , Akerman S . A potent and selective calcitonin gene‐related peptide (CGRP) receptor antagonist, MK‐8825, inhibits responses to nociceptive trigeminal activation: role of CGRP in orofacial pain. Exp Neurol. 2015;271:95–103. 10.1016/j.expneurol.2015.05.005 25981890

[31] Zagami Goadsby AP , Edvlnsson L . Stimulation of the superior sagittal sinus in the cat causes release of vasoactive peptides. Neuropeptides. 1990;16(2):69–75. 2250767 10.1016/0143-4179(90)90114-e

[32] Van Dongen RM , Zielman R , Noga M , Dekkers OM , Hankemeier T , Van Den Maagdenberg AMJM , et al. Migraine biomarkers in cerebrospinal fluid: a systematic review and meta‐analysis. Cephalalgia. 2017;37:49–63. 10.1177/0333102415625614 26888294

[33] Mitchell EV . Serum calcitonin gene‐related peptide (CGRP) concentrations in the horse and their relationship to the systemic inflammatory response. Thesis, Virginia Tech. 2006. p. 1–58.

[34] Joyce CD , Fiscus RR , Wang X , Dries DJ , Morris RC , Prinz RA . Calcitonin gene‐related peptide levels are elevated in patients with sepsis. Surgery. 1990;108:1097–1101. 2247835

[35] de los Santos ET , Mazzaferri EL , Sedra M . Calcitonin gene‐related peptide: 24‐hour profile and responses to volume contraction and expansion in normal men. J Clin Endocrinol Metab. 1991;72:1031–1035. 10.1210/jcem-72-5-1031 2022704

[36] Messlinger K , Vogler B , Kuhn A , Sertel‐Nakajima J , Frank F , Broessner G . CGRP measurements in human plasma – a methodological study. Cephalalgia. 2021;41:1359–1373. 10.1177/03331024211024161 34266288 PMC8592105

[37] Trasforini G , Margutti A , Portaluppi F , Menegatti M , Ambrosio MR , Bagni B , et al. Circadian profile of plasma calcitonin gene‐related peptide in healthy man. J Clin Endocrinol Metab. 1991;73(5):945–951. 1834691 10.1210/jcem-73-5-945

[38] Portaluppi F , Trasforini G , Margutti A , Vergnani L , Ambrosio MR , Rossi R , et al. Circadian rhythm of calcitonin gene‐related peptide in uncomplicated essential hypertension. J Hypertens. 1992;10:1227–1234. 10.1097/00004872-199210000-00017 1335005

[39] Kloock T , Hellige M , Kloock A , Feige K , Niebuhr T . Application of the HRE‐S to 140 horses with trigeminal‐mediated headshaking and the association of clinical signs with diagnosis, therapy, and outcome. Front Vet Sci. 2024;11:11. 10.3389/fvets.2024.1329054 PMC11027008 38645651

[40] Merighi A , Kar S , Gibson SJ , Ghidella S , Gobetto A , Peirone SM , et al. The immunocytochemical distribution of seven peptides in the spinal cord and dorsal root ganglia of horse and pig. Anat Embryol. 1990;181:271–280. 10.1007/BF00174620 1692451

[41] Gibson SJ , Polak JM , Bloom SR , Sabate IM , Mulderry PM , Ghatei MA , et al. Calcitonin gene‐related peptide immunoreactivity in the spinal cord of man and of eight other species. J Neurosci. 1984;4:3101–3111. 10.1523/jneurosci.04-12-03101.1984 6209366 PMC6564846

[42] Story MR , Haussler KK , Nout‐Lomas YS , Aboellail TA , Kawcak CE , Barrett MF , et al. Equine cervical pain and dysfunction: pathology, diagnosis and treatment. Animals. 2021;11:1–21. 10.3390/ani11020422 PMC7915466 33562089

[43] Woodie B , Johnson AL , Grant B . Cervical vertebral stenotic myelopathy. Vet Clin N Am Equine Pract. 2022;38:225–248. 10.1016/j.cveq.2022.05.002 35953144

[44] Ackery AD , Norenberg MD , Krassioukov A . Calcitonin gene‐related peptide immunoreactivity in chronic human spinal cord injury. Spinal Cord. 2007;45:678–686. 10.1038/sj.sc.3102020 17339890

[45] Krenz NR , Weaver LC . Sprouting of primary afferent fibers after spinal cord transection in the rat. Neuroscience. 1998;85:443–458. 10.1016/S0306-4522(97)00622-2 9622243

[46] Gárate G , Pascual J , Pascual‐Mato M , Madera J , Martín MMS , González‐Quintanilla V . Untangling the mess of CGRP levels as a migraine biomarker: an in‐depth literature review and analysis of our experimental experience. J Headache Pain. 2024;25(1):69. 10.1186/s10194-024-01769-4 38684990 PMC11057141

[47] Lindh C , Liu Z , Welin M , Ordeberg G , Nyberg F . Low calcitonin gene‐related, peptide‐like immunoreactivity in cerebrospinal fluid from chronic pain patients. Neuropeptides. 1999;33:517–521. 10.1054/npep.1999.0772 10657534

[48] Madigan JE , Bell SA . Owner survey of headshaking in horses. J Am Vet Med Assoc. 2001;219:334–337. 10.2460/javma.2001.219.334 11497047

[49] Cernuda‐Morollón E , Larrosa D , Ramón C , Vega J , Martínez‐Camblor P , Pascual J . Interictal increase of CGRP levels in peripheral blood as a biomarker for chronic migraine. Neurology. 2013;81:1191–1196. 10.1212/WNL.0b013e3182a6cb72 23975872

